# Comparative analysis of different pit and fissure sealants' marginal sealing abilities

**DOI:** 10.6026/973206300212145

**Published:** 2025-07-31

**Authors:** Keyur Joshi, Saikiran Bahadur, Pavithraa Jayakumar, M. Arul Pari, Ruchi Garg, Mohamed Tharwat Salama, Swagatasree Haldar

**Affiliations:** 1 Department of Pediatric and Preventive Dentistry, GDCH, Jamnagar, Gujarat, India; 2 Departments of Prosthodontics, Springfield Dental, Springfield, Massachussets, United State of America; 3 Department of Pedodontics and Preventive Dentistry, Chettinad Dental College and Research Institute, Kelambkkam, Chennai-603103, Tamilnadu, India; 4 Department of Pediatric and Preventive Dentistry, Sriramchandra Dental College, Porur, Chennai, Tamilnadu, India; 5 Department of Public Health Dentistry, Swami Devi Dyal Hospital and Dental College, Panchkula, Haryana, India; 6 Department of Pediatric Dentistry, Orthodontics and Pediatric Dentistry, College of Dentistry, Qassim University, KSA and Pedodontics and Department of Preventive Dentistry, Sharad Pawar Dental College and Hospital, Datta Meghe Institute of Higher Education and Research, India; 7 Intern, Kalinga Institute of Dental Science, Kalinga Institute of Industrial Technology (KIIT) Deemed to be University, Patia, Bhubaneswar, Odisha, India

**Keywords:** Dental caries, pit and fissure, sealants, ultrasealXT plus

## Abstract

It is commonly known that pit and fissure sealants are efficient at preventing occlusal caries. Therefore, it is of interest to assess two distinct pit and fissure sealants' marginal sealing capabilities. A total of forty human premolar teeth were split into two sealant groups: fissuritfx and ultrasealXTplus sealant (group i). The samples were submerged in a 2% methylene blue solution for a full day following the application of the sealant. An optical stereomicroscope was used to examine the sectioned samples. Better performance was found with ultrasealXT plus compared to Fissurit FX.

## Background:

Dental caries is the most common dental disease and is considered a global public health issue that affects both adults and children. The main factor contributing to the high frequency of dental caries is dietary habits that contain more sugar and carbs. Regular fluoride application is the most tried-and-true strategy for preventing dental cavities [1]. Because of the intricate morphology of their occlusal surfaces, which include pits and fissures, newly erupted primary and permanent molars are particularly vulnerable to dental caries [2]. Tooth decay is facilitated by occlusal pits and fissures, which encourage the stagnation of germs and food debris [3]. Pits and fissures are categorised as V, U, I and K. V and U may be cleaned by themselves and don't require invasive methods, but I and K can't be cleaned by themselves and need to have the defect area sealed [4]. Various preventive strategies are widely used for sealing deep pits and fissures such as pit and fissure sealants [2]. The capacity of sealants to stop the growth of biofilm and its acidic byproducts is what mostly determines their success rate. Sealant retention is mostly dependent on exact technique and perfect isolation. For the process to be successful, the sealant's adherence to the tooth surface is crucial [5]. Despite being thought of as effective preventive measures, failure rates frequently range between 5% and 10% each year, primarily as a result of poor pit and fissure sealant retention [2, 6]. The tooth may be more susceptible to dental caries if there is microleakage at the sealant edges [7]. The marginal sealing ability of the fissures has a significant impact on the effectiveness of pit and fissure sealing materials [1]. In order to produce a satisfactory bond, etching is an essential step in any adhesive procedure [5]. There are various commercially pit and fissure sealant products such as;Ultraseal XT, Helioseal F, Glass Ionomer Cement (type IV), Clinpro, Smart Seal loc F, Embrace Wetbond, Fuji Triage, Tetric Flow, Conseal F, Fissurit FX and sliver nano particle based sealants [1, 8 and 9]. Therefore, it is of interest to assess the marginal sealing ability of two types of commercial pit and fissure sealants.

## Materials and Methods:

The paediatric dentistry department conducted this in vitro investigation. For this investigation, 40 human premolar teeth that were removed for orthodontic purposes and were free of pathology were used. Following extraction, every tooth was cleaned of any remaining debris, disinfected with a hydrogen peroxide solution, and then kept at room temperature in artificial saliva. A rubber cup in a slow-speed contra-angle handpiece was used to clean all of the tooth's fissures for 15 seconds using aqueous slurry of 5g pumice and 4ml water. Then teeth were rinsed with air-water spray and stored in saline. Total 40 and they were alienated into 2 equal groups of 20 samples each as; Group I- application of ultrasealXT plus (Ultradent, USA) sealant having 42.7 shear bond strength and Group II- application of Fissurit FX (Voco, German) sealant with 6.2 shear bond strength. For 20 seconds, teeth in both groups were etched using a 37% phosphoric acid gel. After 20 seconds of water rinsing, they were gently dried using a three-way syringe. Upon ocular inspection, the teeth showed a consistent frosty appearance. After etching, each tooth samples occusal fissures were sealed with respective fissure sealants as per manufacturer instructions followed by light curing. After that, every tooth in both groups was imbedded in acrylic resin, thermocyclized for 500 cycles at 5°C, 37°C, and 55°C with a 30-second dwell time, and then preserved in artificial saliva. The study was done by trained investigator. Then samples were immersed in 2% methylene blue solution for 24 hours. Later the samples were sectioned and examined under an Optical Stereomicroscope for dye penetration to check microleakage and sealing ability of fissure sealant. The following was the grade for the microleakage: Grade 0: No dye penetration, Grade 1: Dye penetration over one-third of the entire length of the sealant-tooth structure interface, Grade 2: Dye penetration between one-third and two-thirds of the total interface length, and Grade 3: Dye penetration over two-thirds of the total interface length. Using SPSS Version 23.0, the collected data was statistically assessed using the Mann Whitney U test at p<0.05 and Chi square.

## Results and Discussion:

It was observed that five samples (25%) in Group II showed Grade 0 dye penetration, whereas ten samples (50%) in Group I had no dye penetration at all. In 6 out of 20 samples (30%), Group II had the greatest dye penetration (Grade 3) (p=0.01) (Table 1, 2 - see PDF). Because pits and cracks retain plaque; they are more difficult to clean [10]. Self-adhering flowable composites are the result of recent advancements in restorative dentistry. Dental caries is more likely to occur in tooth pits and fissures. It has been demonstrated that using a sealant is an economical and efficient way to stop children's fissure caries [11, 12]. By creating a physical barrier that stops cariogenic germs from colonising, the sealants help to prevent dental cavities. Numerous standard parameters including penetration coefficient, microleakage, tensile bond strength, sealant viscosity, and length of resin tag created; influence how successful pit and fissure sealants are [1]. In the current research we found that, UltraSeal XT plus were better with lesser microleakage compared to Fissurit FX sealant. The temperature range used in this investigation was 5°C to 55°C, which was deemed to be the most clinically relevant by Penugonda *et al.* in a number of their studies [13]. Similar to our result, Sridhar *et al.* assessed the marginal sealing capability of variouspit and fissure sealants and found better performance of Clinpro as compared to Helioseal-F [1]. Wadhwa *et al.* concluded that, the marginal integrity of Dyad Flow was considerablyenhanced than that of Helioseal-F [11]. According to Suryavanshi *et al.* the flowablegiomer had the most microleakage, whereas the Gic Fuji VII had the lowest [14]. According to Demirel *et al.*'s research, glass ionomer-based Fuji IX-GP and giomer-based Beauti-Sealant could be utilised as substitutes for sealants made of resin [15]. Garg*et al.* assessed the self-etching's adaptability and sealing power in comparison to traditional pit and fissure sealants. They came to the conclusion that self-etching sealants are comparable to traditional acid etch sealants in terms of microleakage, sealant penetration, and adaptability [5]. Joshi *et al.* evaluated the sealing capabilities of three distinct pit and fissure sealants and came to the conclusion that composite material outperformed glass ionomer cement and compomer as a sealant material [4]. According to Mascarenhas *et al.* [16] and Boksman *et al.* [17], applying a bonding agent prior to sealing does not enhance sealant retention over time. According to Bartaria *et al.* nano-hybrid flowable composite outperformed the others in terms of penetration depth [10]. Quiroga *et al.* discovered no appreciable variations in the marginal seal or microleakage between the standard sealant and the sealant incorporating silver nanoparticles [8]. The sealant's resistance and adhesion are unaffected by the addition of AgNPs. Composite resin is the most often used substance for dental sealants, though they can be constructed of many different materials. The application of nanotechnology to flowable composites enables the creation of a composite that preserves the elasticity, adaptability, and advantageous handling properties of the flowable composite [10]. The retention rates of filled and unfilled sealants did not differ statistically significantly, according to Bagheri2022 [18] and Alsabek 2021 [19]. According to Alirezaei *et al.* resin-based FS had a statistically significant greater retention rate than GIC [20]. To enable penetration into the etched enamel's microcracks, the sealant material needs to have a high degree of wettability and viscosity [21]. This property is shown by "coefficient of penetration. Better results were found in present study with UltraSeal XT plus due to greater shear bond strength and sealing ability compared to Fissurit FX. According to Rani *et al.* self-adhering flowable composite outperform traditional fissure sealant in terms of shear bond strength and marginal sealing performance [22]. The limitation of the present study is smaller sampling size and it was in vitro evaluation. Further studies are needed to validate the result with larger sample size with clinical study.

## Conclusion:

UltraSeal XT plus performed significantly better than Fissurit FX for microleakage ratings.
